# Exploring utilisation of the allied health assistant workforce in the Victorian health, aged care and disability sectors

**DOI:** 10.1186/s12913-021-07171-z

**Published:** 2021-10-23

**Authors:** J. Huglin, L. Whelan, S. McLean, K. Greer, D. Mitchell, S. Downie, M. K. Farlie

**Affiliations:** 1 Allied Health Workforce Innovation, Strategy, Education & Research (WISER) Unit, Monash Health, 400 Warrigal Road, Cheltenham, VIC 3192 Australia; 2 Faculty of Medicine, Nursing and Health Sciences, Monash University, 27 Rainforest Walk, Clayton, VIC 3168 Australia; 3 Department of Health, Victoria State Government, Lonsdale Street, Melbourne, VIC 3000 Australia

**Keywords:** Allied health, Assistant, Support, Workforce, Career, Delegation

## Abstract

**Background:**

Allied health assistants (AHAs) support allied health professionals (AHPs) to meet workforce demands in modern healthcare systems. Previous studies have indicated that AHAs may be underutilised in some contexts. This study aims to identify factors contributing to the effective utilisation of AHAs across health, aged care and disability sectors and possible pathway elements that may optimise AHA careers in Victoria.

**Methods:**

Using an interpretive description approach data collection included a workforce survey and semi structured interviews (individual and group). Data analysis included descriptive statistics, independent t-tests and thematic analysis. Participants included allied health assistants, allied health professionals and allied health leaders in the health, aged care or disability sectors; educators, managers or student of allied health assistance training; and consumers of Victorian health, disability or aged care services.

**Results:**

The literature scan identified numerous potential barriers to and enablers of AHA workforce utilisation. A total of 727 participants completed the survey consisting of AHAs (*n* = 284), AHPs & allied health leaders (*n* = 443). Thirteen group and 25 individual interviews were conducted with a total of 119 participants. Thematic analysis of the interview data identified four interrelated factors (system, training, individual and workplace) in pre-employment training and workplace environments. These factors were reported to contribute to effective utilisation of the AHA workforce across health, aged care and disability sectors. Study findings were also used to create a conceptual diagram of potential AHA career pathway elements.

**Conclusion:**

This study identified pre-employment and workplace factors which may contribute to the optimal utilisation of the AHA workforce across Victorian health, aged care and disability sectors. Further study is needed to investigate the transferability of these findings to national and global contexts, and testing of the conceptual model.

**Supplementary Information:**

The online version contains supplementary material available at 10.1186/s12913-021-07171-z.

## Background

The modern healthcare system faces multiple global challenges related to population ageing, increasing chronic disease burden and projected workforce shortages [1, 2]. The allied health professions have demonstrated leadership in the development of flexible workforce models [3, 4], for example the engagement of allied health assistants (AHAs) to support the allied health professionals (AHPs) to enable efficient allocation of workforce resources [5–7]. AHAs work under the delegation and supervision of AHPs to support the delivery of allied health services and may work with one or several allied health therapy disciplines [8] and in a variety of settings, including health, aged care and disability services [9]. AHAs carry out delegated clinical and non-clinical tasks, allowing AHPs to focus on the complex clinical work that requires their expertise [10–12]. AHA duties may include providing individual therapy sessions, delivering therapy groups, equipment set-up and administrative tasks [9].

Previous studies indicate that AHAs may be underutilised in some contexts. A workforce utilisation study of Australian public health and community settings reported that AHPs spent a proportion of their time in metropolitan (11%) and rural/remote (17%) contexts performing tasks that could have been delegated to an AHA [13]. Similarly, a follow-up study in metropolitan community and ambulatory care found that AHPs spent 24% of their time performing tasks that could have been delegated. AHA workforce availability was a key limitation to delegation of tasks identified in these settings [13]. Other barriers to the optimal utilisation of the AHA workforce include indistinct role boundaries and professional protectionism [9]; lack of defined scope of practice, competency standards and structures for AHAs [14, 15]; AHP reluctance to delegate clinical tasks [8], and assumptions that AHPs understand good delegation practice [16]. Variation in role descriptions, the extended time required for an AHA to reach a proficient skill level and AHA training limitations have also been identified as barriers to optimal utilisation of AHAs [8].

Victoria is the second most populous state in Australia, with a population of approximately 6.6 million. In Victoria, AHAs are employed in the health, disability and aged care settings. Various tools have been developed by the Victorian Department of Health to guide employers of AHAs to support this workforce in a devolved governance model. These tools include examples such as the Victorian Assistant Workforce Model (VAWM) [17] and two supervision and delegation frameworks, one for the health [12] and one for the disability sector [18]. However, the utilisation of these tools is not universal, and it is unclear what else may support the optimisation of the AHA workforce in the Victorian context.

The aims of this study were to examine factors that promote the effective utilisation of AHAs across various sectors, and identify potential factors that could support optimal AHA utilisation, governance, education and training requirements in Victoria. The research questions for this study were:

1. What do key stakeholders identify as factors contributing to the effective utilisation of AHAs across health, aged care and disability sectors?
2. What are the possible pathway elements that may optimise AHA careers in Victoria?

## Methods

### Study design and research team

The design for this study was a mixed method study design using an interpretive description framework. Interpretive description is an applied pragmatic qualitative methodology that allows for the exploration of individual and shared experiences across contexts [19, 20]. The epistemological stance of this study is situated in interpretivism [21]. Interpretive description has particular applicability in the investigation of qualitative research questions intending to inform practice change [19, 20, 22] and has previously been applied to healthcare workforce research in nursing and allied health [23, 24] Interpretive description is not limited to a particular methodological process, rather study design and data collection methods are grounded in an analytical framework informed by the nature and scope of the research question/s [19, 20].

This study followed an iterative process commencing with a review of barriers to and enablers of the optimal utilisation of the AHA workforce identified in existing literature. A survey informed by the literature scan presented questions to key stakeholders ‘in the field’ to explore their applicability in the Victorian context. Literature scan and survey results were subsequently used to develop question guides for individual and focus group interviews that further explored the research questions with a particular focus on how AHA workforce utilisation could be optimised in health, aged care and disability sectors [19, 20].

A reflexivity exercise at the outset of this study established the positions of individual team members with an extensive range of knowledge and experience in allied health professional and assistant clinical practice (physiotherapy and occupational therapy), education and training, as well as operational, governance and policy leadership, and qualitative research. All AHP members of the research team had extensive experience working with AHAs in clinical, managerial and strategic contexts, and one member of the team was working as an AHA (SM). The research team occupied both ‘insider’ and ‘outsider’ positions [25, 26] and brought rich and nuanced perspectives on the research questions into the project context. The research team consulted regularly with a project steering committee to inform data interpretation. The steering committee was representative of key sector stakeholders from healthcare, disability, aged care, education and professional associations. Committee membership ranged from 29 to 33 individuals during the study. Reporting of study findings was informed by the Consolidated Criteria for Reporting of Qualitative Research (COREQ) [27]. Ethical approval was granted by the Monash Health Human Research Ethics Committee (RES-20-0000-356 L / ERM 64899).

### Participants

Participant sampling included a combination of population (survey phase) and purposive (individual and focus group interview phase) sampling. To be eligible participants had to be currently employed in the state of Victoria, Australia as either an: AHA, AHP or allied health leader in the health, aged care or disability sectors, or an educator, manager or student of allied health assistance training in the Vocational Education and Training (VET) sector, or a consumer of Victorian health, disability or aged care services. The health sector was inclusive of public and private hospitals and community and mental health settings. The disability and aged care sectors were inclusive of residential and community care settings. There were no restrictions placed on individual participation in either or both the survey and interview stages of the study and repeat interviews were not carried out. Individuals were excluded if they did not have access to the required technology to participate (email, online survey – survey phase or teleconferencing – individual and focus group interview phase).

### Sources of data and data collection

#### Literature scan

A literature scan was conducted in March 2020 by a single author (KG). Databases searched were Medline, ProQuest, Embase and Joanna Briggs Institute from the year 2000 to March 2020. Sources of grey literature included publicly available government and allied health professional association reports identified online. Search terms included: allied health assistant or assistant for each allied health discipline, combined with: allied health workforce, workforce planning, workforce redesign, workforce utilisation, governance, delegation, supervision, scope of practice, education, professional development, professional acceptance and inter-professional practice. Records included were limited to English language, peer reviewed articles, commentaries and reports.

#### Scoping survey of key stakeholders

A search for existing surveys did not identify a survey directly addressing the research questions, therefore a survey was constructed following the recommendations of DeVellis [28]. Steps in survey construction included: a) drafting of demographic items and items that incorporated barriers and enablers identified in the literature scan, b) consideration of response options with a combination of categorical and Likert scale questions included, c) survey structure and question wording review provided by a subject matter expert in survey construction, d) survey pilot testing for relevance, clarity and utility by a small representative group of project steering committee members (*n* = 5) prior to e) survey finalisation. The survey included ten demographic questions, and one categorical nomination matrix with 19 items (See Supplementary Table 2, Additional file 1) and two Likert scale matrices with ten and five items respectively (See Supplementary Table 3, Additional file 1). The finalised survey was administered electronically via a secure online platform and was open for 6 weeks. A population sampling approach was used with an email invitation sent to all Victorian health, aged care and disability organisations through state-wide distribution lists, the project steering committee, social media channels, and health professional networks.

#### Semi-structured interviews – individuals and focus groups

Construction and conduct of interviews was consistent with the methods described by Liamputtong [29, 30] and McGrath and colleagues [31]. Purposive and quota sampling methods were used to recruit participants to provide perspectives from metropolitan and regional Victorian health, aged care, and disability and VET settings. A sampling grid was used to map group selection and quotas [32]. Sampling quotas were used to ensure representation of AHA and non-AHA participants represented in interviews, as well as participants from metropolitan, rural and regional settings, and health, disability, aged care and education sectors (See Supplementary Table 4, Additional file 1). Invitations to participate were distributed by the same email method used to distribute the survey. Consumer participants were identified through personal networks of steering committee members.

Survey findings were explored in 38 interviews (focus groups *n* = 13, individuals *n* = 25). Interviews were conducted by pairs of research team members (LW/KG; DM/KG; SM and KG or LW) with participants and no other attendees via video-conference using a semi-structured question guide. All interviewers were experienced in conducting interviews and facilitation of focus group discussions. Focus groups were organised in five stakeholder groups: AHAs, AHPs and allied health leaders, AHA certificate training program educators and managers, AHA certificate training program students and consumers of Victorian, health, disability and aged care services. To limit the potential impact of power imbalances between AHA and non-AHA participants, these groups were interviewed separately. Interviewer allocations were managed to ensure no direct managerial relationships between interviewers and participants. Interviews commenced with a summary of the research study purpose and the interviewer’s interest in the research topic. All audio-recordings were transcribed verbatim by a professional transcription service and checked for accuracy and de-identified by an interviewing member of the research team (KG) prior to team analysis.

### Data analysis

An annotated bibliography was used to organise the literature scan results. Directed content analysis was used to chart and synthesise the data [33]. The research team developed the analysis framework based on the research aims, which was then applied to each included record to identify barriers and enablers in four categories: governance, training & development, context and professional relationships. The findings were conceptualised diagrammatically (See Supplementary Figure 1, Additional file 1). Survey data was analysed using descriptive statistics for continuous data such as means and standard deviations, and for categorical data such as medians and interquartile ranges. AHA and non-AHA responses, including Likert-type item responses [34], were compared with independent t-tests. For comparisons of binary responses Levene’s test of Equality of Variances was used to determine the appropriate output to report [35]. A Bonferroni correction was applied to adjust for multiple comparisons [36]. All statistical tests were conducted in SPSS [37].

Interview data was analysed using thematic analysis as described by Miles and colleagues [38]. Analysis steps included data immersion, familiarisation, coding, display and categorisation. The coding process was a combination of provisional (deductive) coding based on the literature scan and survey results, and descriptive (inductive) open-coding to develop the coding framework. Three researchers (KG, MF, LW) independently coded a sample of the data (2 focus group and 2 individual interview transcripts) to develop the initial coding framework. The same researchers completed the coding as a team by referencing and building on the coding framework by continuous re-examination of the data for patterns and themes within and across participant categories and contexts [38]. Three researchers (KG, MF, LW) met regularly during the analysis process to discuss and refine the analysis and to devise the network diagram. Two researchers (LW, KG) then engaged in multiple rounds of consultation with the project steering committee and reviewed relevant industrial agreements and existing workforce planning tools, such as the Victorian Assistant Workforce Model (VAWM) [17]. All data was then reviewed with a third researcher (MF) to inform the creation of a diagram to conceptualise and interpret the findings [19, 38]. Qualitative analysis was conducted in NVivo12 [39].

## Results

### Literature scan

The literature scan identified numerous potential barriers to and enablers of AHA workforce utilisation (See Supplementary Figure 1, Additional file 1). These included diversity of AHA roles [40, 41], inconsistency in AHA skills and capabilities [8, 9, 40, 42], service requirements [41], workforce capacity [13], gaps and inadequacies in governance and training of AHAs and AHPs [9, 43, 44].

### Survey results

There was a 91% completion rate for eligible participants who commenced the survey. A total of 727 participants completed the survey consisting of AHAs (*n* = 284), AHPs & allied health leaders (*n* = 443). Demographic details of the respondents are reported in Supplementary Table 1a and Supplementary Table 1b, Additional file 1. The survey respondents identified potential barriers and enablers of AHA utilisation. AHA respondents were significantly more likely than AHP & allied health leader respondents to nominate the following skills as core AHA work: *confidence, assertion of boundaries, drive, self-development of role*, *assertiveness, thinking outside the box, self-direction, experience, willingness to accept responsibility and clinical competence* (*p* < 0.002) (See Supplementary Table 2, Additional file 1). AHP & allied health leader respondents were significantly more satisfied than AHA respondents with most job role elements presented: *upskilling options, access to continuing professional development, leading and involvement in quality improvement, leading and involvement in research, involvement in student education, stimulation in role and career opportunities* (*p* < 0.003) (See Supplementary Table 3, Additional file 1). AHAs also agreed more than AHP & allied health leaders that *AHAs were optimally utilised, and that formal and informal clinical supervision needs were adequately met* in their workplace (*p* < 0.01) (See Supplementary Table 3, Additional file 1).

### Semi-structured interviews – individuals and focus groups

The profile of the thirteen focus groups and 25 individual interview participants is presented in (See Supplementary Table 4, Additional file 1) with a total of 119 participants. No participants withdrew from the interviews. Average interview duration was 1-h. Data analysis commenced from the first interview, and structured post-interview debriefing notes completed by the interviewers were used by the research team to inform analysis and confirm that no new information was being garnered from interviews and the team was satisfied information power had been achieved before the interview stage was finalised [45].

#### Part 1: thematic analysis and network mapping

**Fig. 1:**
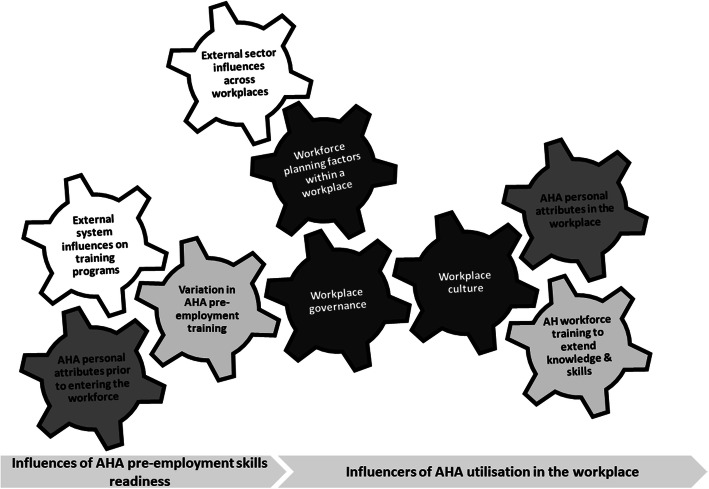
Network map of pre-employment and workplace influences on optimal utilisation of the AHA workforce. This map illustrates the relationship of pre-employment training and workplace related themes (grey arrow bars). The relationships between themes were categorised into system factors (white cogs), training factors (light grey cogs), individual factors (dark grey cogs) and workplace factors (black cogs)

Thematic analysis of the interview data identified a number of interrelated factors that contributed to the promotion of effective utilisation of AHAs across health, aged care and disability sectors. An illustration of the analytic process from raw data to theme and category is presented in (See Supplementary Table 5, Additional file 1). Identified themes were represented in a network map that illustrates the influences related to both pre-employment and post-entry to the workforce (Fig. 1). Thematic descriptions follow in the categories of individual, systems, workforce, and training factors.

#### Individual factors

Two themes were categorised as individual factors: (1) AHA personal attributes prior to entering the workforce and (2) AHA personal attributes in the workplace. These related to aptitude required for an AHA career, requisite knowledge and readiness for employment, along with attributes and skills in the workplace. Participants reported these factors influenced whether an individual was suited to and thrived in the AHA role. In particular, participants indicated intrinsic personal attributes played a key aspect in shaping skills readiness and success in work roles.

*I’ve had to prove my competency, capability, reliability etc. The teams had not had … great experiences with AHAs in the past, so I had to change their mindset. I had to gain their trust and show them I’ve got the skills and knowledge to assist them. I was working below my capabilities but now I’m working at my full potential which is good. I’ve shown the teams that I’m more than capable.”* [Focus Group: AHA - metro]

Participants identified core skills and attributes necessary for success in the workplace that were primarily related to an AHA’s personal capabilities. For example, an AHA with awareness of their scope of practice, an ability to recognise and report clinical risk and the confidence to speak up around their capabilities and training needs was seen as more likely to succeed in the role. Participants identified the ability to learn and develop new skills, and manage competing demands were also essential.

*“.. as long as they [AHAs] have got the skills to respond, learn, be adaptable, show good initiative, they are the key things that you look for … you’re more than likely going to have to train them [AHAs] up to the specific tasks that you want them to do anyway.”* [Focus Group: Allied health leader – regional]

#### System factors

Two themes were categorised as systems factors: (1) External system influences on training programs and (2) External sector influences across workplaces. These related to training funding models, industrial agreements, geographical locations of services and emerging health issues. For example, at a sector level the Certificate IV in Allied Health Assistance was reported as a suitable qualification to work in Victorian health and community services, however there was uncertainty expressed about the suitability of this qualification in catering to the current needs of the disability and aged care sectors.

*“ … the Certificate IV Allied Health Assistance … [would benefit from] … different subjects like healthcare, NDIS and a few other things to help shape it so allied health assistants that are restarting or getting into the course are able to learn … and develop their role.”* [Focus Group: AHA – metro]

Participants reported the availability of funding influenced both the uptake and planning of the AHA workforce. Preferential funding of the allied health professional workforce in the health sector, and an overall lack of funding for any allied health workforce in the aged care sector were identified as key barriers to systematic utilisation of AHAs.

*“We know they (AHAs) are effective and so … there’s a lot of work still going in to showing their effectiveness even though we know they’re effective. I think the government are very happy to model or provide funding for allied health but I see AHAs just as important as your dietetics, your physio, your OT. There needs to be provision for AHA workforce as well in that big picture thinking from the Department of Health.”* [Focus Group: Allied health leader – regional]

In contrast, disability sector participants reported expansion of the AHA workforce, which they attributed to the opportunities arising from the funding available from the National Disability Insurance Scheme (NDIS).

*“There are a couple of peak bodies in our sector who are really pushing for the use of AHAs … that’s the way the NDIS is wanting to go.”* [Focus Group: Allied health leader – metro]

Allied health leaders representing professional associations expressed a diversity of views about their association’s priorities toward the AHA workforce. Some supported utilisation of the AHA workforce via the provision of educative resources for their constituent members, while others reported protecting professional workforce funding.

*“ … an important message we should be promoting, is [that] an AHA can actually increase your capacity, but it’s not to replace the professionals.”* [Individual: Professional association representative – metro]

*“Rather than looking at growing the pie, it’s basically how much of the pie can I keep for myself”* [Individual: Professional association representative – metro]

#### Workforce factors

Three themes were categorised as workforce factors: (1) workforce planning within a workplace; (2) workplace culture and (3) workplace governance. These included workforce models, supervision and delegation structures, formal credentialing and AHA representation. Participants identified advantages to using AHA workforce models, such as fostering consumer centred care and a more efficient use of resources when planning and redesigning the workforce.

*“ … I think our AHAs, from a patient or client perspective, if they can get more of their needs met by one person you end up with a better relationship and I think [AHAs] can actually knit together the professions as well … ”* [Individual: Allied health leader– metro]

AHA workforce planning was largely reported as ad hoc and appeared to be driven by high demands for AHP services, new service opportunities or when undertaking a service evaluation. Factors that influenced the uptake and utilisation of the AHA workforce included the utilisation of allied health workforce frameworks to inform AHA practices, the appropriateness of supervision structures, clarity of grading structures and scope of practice for AHA roles. AHAs reported that AHPs were not clear about the role scope of a grade-two compared to a grade-three AHA.

*“ … there needs to be a clear understanding what a grade-two and a grade-three [AHA] does because I sometimes do a grade-three role but I’m employed as a grade-two and then every time I say to my supervisor, “I’m a grade-two, this is what I can do,” she’s like, “But you’ve been here for a while, you can do this.”* [Focus Group: AHA – metro]

Participants reported that workplace culture influenced the utilisation of AHAs by shaping how they were viewed within a team, and how well AHAs were supported to learn and develop. Participants reported an inclusive and respectful culture supported collaborative and mutually beneficial relationships between AHPs and AHAs.

*“It’s like a cohesive work environment where everybody’s work is considered to be equally important matters because then, you will help them and they will help you … - and it just works better as a team. It’s quite hard sometimes to assert yourself … and say, “Well, what I do for this patient is just as important as what you do,” and so … the AHA role needs to be recognised as part of that whole team.”* [Focus Group: AHA – metro]

*“It’s about that mutual respect and I think really reinforcing that they’re [AHAs] a really incredibly valuable part of the team and they often bring brilliant insights. And I know in our unit, they always know their patients so well and so intimately. [AHAs] bring brilliant suggestions and ideas and thoughts about how things could be done differently.”* [Focus Group: Allied health leader– metro]

The supervisory relationship between an AHP and AHA were seen to affect how AHAs were supported and progressed in their role, which was influenced by an AHP’s supervisory experience.

*“If we have a good clinical governance, we have good supervision structures, that’s supporting the AHAs and their needs and what gaps that there might be, as well as addressing issues around health professionals’ confidence in working with AHAs.”* [Focus Group: AHP – metro]

A dedicated role to professionally oversee and advocate for the AHAs was seen to influence the development of the workforce, and supervision and delegation practices. There was a perception that effective delegation was seen to require clear processes and good communication. This was evident particularly where AHA roles varied across settings which magnified the importance of the orientation of AHPs to individual AHA roles.

*“We have a PowerPoint presentation [for] brand-new staff … in their first week that explains the role of the AHAs in the department and what [AHA] scope of practice is and what [AHAs] can do … I think it helps if it comes from the AHAs because you can explain your role much better, and it helps to just give [AHPs] an understanding of what we can do.”* [Focus Group: AHA – metro]

#### Training factors

Two themes were categorised as training factors: (1) Variation in pre-employment training and (2) Allied health workforce training to extend knowledge and skill. These included VET courses offered, AHA student placements, workplace orientation and AHA on-the-job competency-based training and professional development. Variation in the content and delivery of the Certificate III and IV in Allied Health Assistance course curriculum was identified as a factor influencing AHA student skill development. A shortage of suitably experienced VET sector teachers, focus on a limited number of allied health professions and limited clinical exposure during AHA certificate training was also seen to leave graduates poorly prepared for work in some settings.

*“There’s probably not enough placement time on the certificate [course] to fit in enough experience, and also if you work in a small discipline these days the certificate course is only geared towards physio and OT. “* [Focus Group: AHA – metro]

In the workplace, the level of training provided to AHAs and AHPs influenced utilisation of AHAs. Training included understanding role scope, delegation effectiveness, working relationships and AHA development was perceived as useful. Participants reported that documented competency-based training and assessment programs positively influenced AHA utilisation. This was seen to both improve AHP confidence in an AHA’s abilities, and AHA self-confidence.

*“that’s where we find there are barriers to using AHAs, because sometimes allied health professionals aren’t confident in that supervision and delegation space, and that’s something we’re having to constantly try and develop the skills in our allied health professionals to help support, supervise, develop the AHA. So, I think that’s an area that at our health service we probably need to do some more work in.”* [Focus Group: Allied health leader– metro]

#### Part 2: conceptualisation of AHA careers pathways to optimise workforce utilisation

**Fig. 2:**
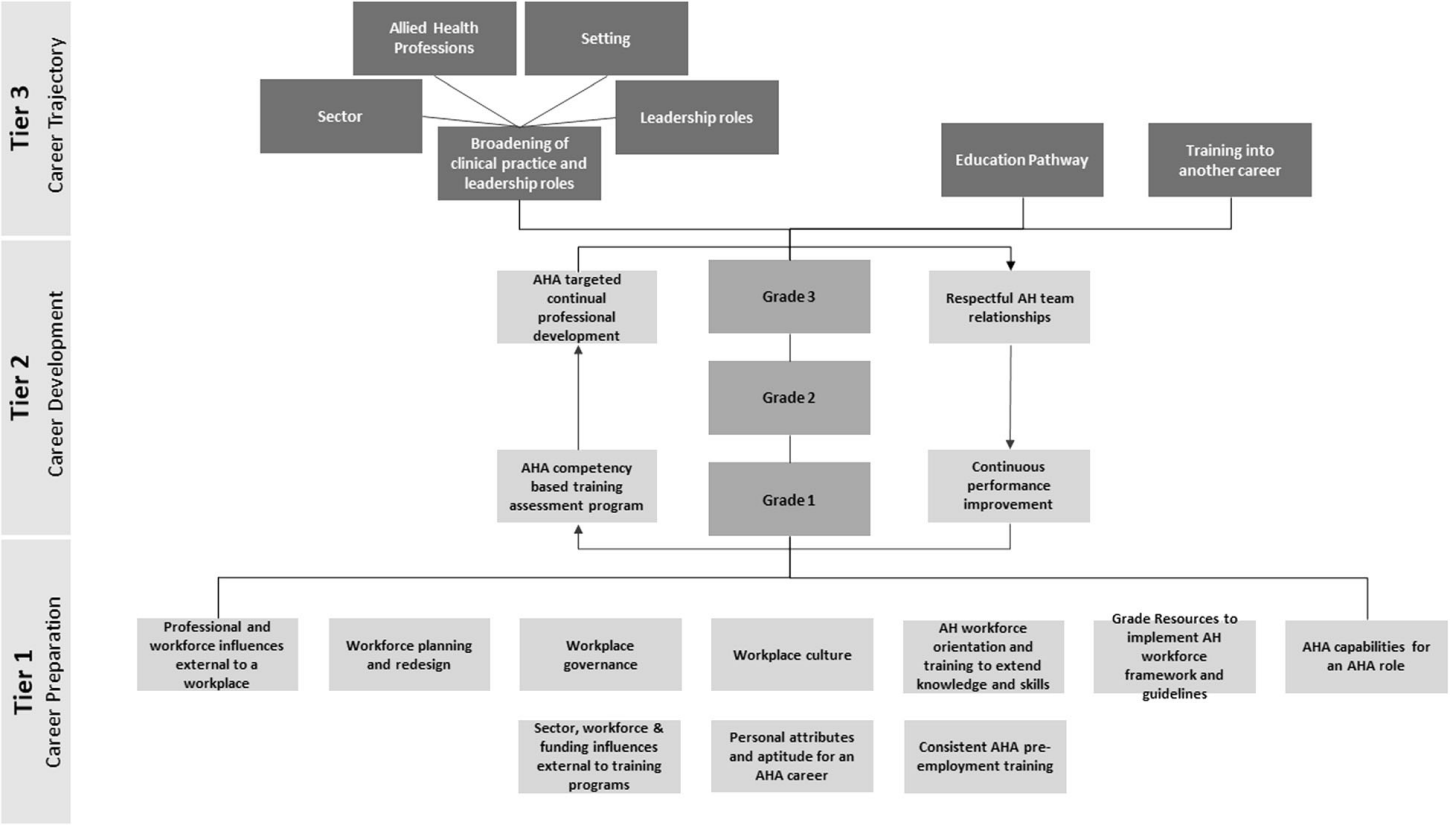
Conceptual diagram of potential AHA career pathway elements to optimise AHA utilisation. This diagram depicts three tiers that conceptually represent stages of an AHA career in Victoria across health, aged care and disability sectors. Tier 1 depicts influences on career preparation. The solid lines indicate systems factors and dotted lines indicate personal factors. Tier 2 shows how ongoing development and workplace culture (light grey boxes) supports progression through the Victorian AHA grade classifications (grey boxes). Tier 3 highlights the breadth of possible development opportunities that may be considered to optimise AHA career trajectories (dark grey boxes)

The network map illustrating pre-employment training and workplace environments that influenced the optimal engagement of AHAs (Fig. 1) was used to create a conceptual diagram of potential AHA career pathways. The research team and the project steering committee engaged in multiple rounds of consultation to devise this interpretive description in the form of the conceptual diagram presented in Fig. 2. This diagram theorises key pathway elements that may lead to optimal utilisation, governance, education and training of the AHA workforce in Victoria.

## Discussion

This study has identified perceived factors that contribute to the promotion of effective utilisation of AHAs across health, aged care and disability sectors. It presents a conceptual interpretation of the possible pathway elements that may optimise AHA utilisation, governance, education and training in Victoria.

### Factors identified by key stakeholders contributing to the promotion of effective utilisation of AHAs across health, aged care and disability sectors

The thematic analysis presented in our network map (Fig. 1) identified a number of barriers to and enablers of optimal utilisation of AHAs across all sectors. These themes were consistent with those previously identified including: a) role definition and defined scope of practice [8, 14, 15, 46, 47]; b) AHP understanding and execution of effective delegation practice [8, 13, 14, 16, 43]; and c) AHA training variations [8, 48, 49].

Key themes identified in this study included the variation in pre-employment training, workforce planning and the impact of workplace culture. Participants described variable pre-employment AHA training, which results in an inconsistent baseline from which industry can recruit, develop and expand the assistant workforce. Policies such as the recent national government initiative to provide free tuition for VET courses were seen to alter the profile of students accessing the certificate in allied health assistance including in Victoria. Non-standardised pre-employment training has also been identified as key issue impacting recruitment and retention of the support workforce in other contexts such as New Zealand [50] and the United Kingdom [51].

Workforce planning across sectors was characterised by underutilisation of existing workforce planning tools, such as the VAWM [17] reported by both survey and interview respondents. This is consistent with previous descriptions of individual profession-led approaches to workforce planning characterised by health professionals basing support workforce planning decisions on hierarchical models of care [52]. In contrast, manager-led approaches base workforce planning decisions on client/service needs and strategic professional role substitution with resource allocation guided by structured frameworks [4, 52]. Factors described by participants in this study that are consistent with support for a managerial approach to the optimisation of the allied health workforce included cost benefit analyses, clear descriptions of delegable tasks and scope of practice, and promotion of consistent evidence-based supervision and delegation. Potential benefits of taking this approach may be increased value placed on the AHA role in the workplace and a shift in culture that supports the inclusion of AHAs in workforce planning decision making.

Benefits of valuing the AHA workforce likely include improved delegation practices, supervisory relationships, consumer and service outcomes. One barrier to AHA workforce utilisation identified in the literature scan was a perception of AHPs that their role may be devalued by optimising the use of the AHA workforce. This finding was not supported by the interviews in this study, where instead a lack of knowledge of the potential scope of AHA role was identified as a barrier to utilisation. However, examples of sub-optimal workforce culture reported by AHA and non-AHA participants were consistent with our literature scan findings and included characterisations of AHAs being treated like ‘second class citizens’ in healthcare teams.

Previous studies have linked a lack of recognition of support worker identity to sub-optimal delegation practices by health professionals with an impact on patient outcomes [15, 53, 54]. The historical hierarchical structure within large health settings may translate into AHAs being positioned at the ‘bottom of the pecking order’, unless local teams make visible efforts to include and value them. Supportive workplace culture was described by AHA and non-AHA participants in this study as a characteristic of organisations with dedicated AHA governance structures, such as leadership positions responsible for the support workforce, and visible AHA leadership with professional oversight (i.e. a seat at the table). This also included well supported clinical supervision and delegation processes, time for AHAs and AHPs to work together on skill development in addition to formal competency and professional development opportunities for AHAs. These findings reflect a broader shift both nationally and internationally towards recognising the support workforce as integral and valued members of care teams [50, 51, 53].

### Pathway elements that may optimise AHA careers in the Victorian context

A range of tools are already available to support planning and development of the AHA workforce in Victoria (e.g. Victorian Assistant Workforce Model [17] and the Credentialing, Competency and Capability Framework [55]). The survey and interview findings in this study indicate that these frameworks are under-utilised and highlighted the influence of individual relationships on AHA workplace utilisation. In other words, our findings suggest that while tools are designed to function at a system level, local culture and individual relationships influence whether the tools are used and how they are implemented. This was further demonstrated by AHA descriptions of role satisfaction being dependent on the individual AHPs they worked with and when working in a team that valued their role.

This study has provided new insights into factors to consider in the future development of AHA career pathways to complement existing tools. AHA and non-AHA interview responses showed more agreement than the survey findings when considering the limitations AHAs experience with regards to accessing CPD and supervision. Interviewees also reported more consistently that soft skills were important pre-requisites for an AHA role, and technical skills could be learned in the workplace. Some existing system level mechanisms that support AHA career pathways include local industrial agreements including the progression of AHA roles from unqualified (Grade 1) to a qualified (Grade 2) upon completion of vocational certificate training. Qualified AHAs may be eligible to apply for one higher classification (Grade 3) with additional experience. Currently, there are no higher role classifications in the Victorian setting, although a fourth grade is included in classifications in other states of Australia. Our findings indicate that personal attributes, pre-employment training options, on the job training and development, workplace operations and governance, and external workplace influences need to be considered at an individual and system level when looking to optimise career pathways for the AHA workforce.

Survey and interview results consistently indicated that AHAs experienced reduced access to career advancement opportunities in comparison to their AHP colleagues. Outside formal role classifications, AHA participants in this study described their experiences in teaching and leadership roles as important pathways to career advancement. Other pathways included access to clinical and leadership opportunities, such as responsibility for quality improvement activities and AHA student education in workplace and vocational education settings. Participants in this study expressed a desire for training and targeted professional development programs to broaden experience, maintain interest, boost leadership capabilities and accelerate entry into vocational teaching and allied health professional training programs. Research in the United Kingdom has previously described a ‘career escalator’ model for incremental development of the podiatry assistant workforce with an emphasis on progression from a support worker to an allied health professional role [56].

While progression from AHA to AHP is one important career path to consider, the findings in our study have also provided new insights into career development opportunities within the AHA role and the importance of a culture of learning. This finding is consistent with research on assistant workforce career development in pharmacy [57, 58] and nursing [59] that have been referred to as ‘career ladder’ programs. Morgan and Conrad describe a program in a subacute long-term care facility training the nursing assistant workforce to develop skills and capabilities that allowed registered nurses to complete higher level clinical tasks [60]. Similarly, a program set in residential aged care had a focus on the improvement of clinical and interpersonal skills, succession planning and career development within the nursing assistant role through an embedded a learning culture and broadening capabilities within scope [60].

### Study rigour and limitations

This study contained several design features to ensure the rigour of the study findings. The research team included AHA and AHPs with clinical, research, management and policy experience, who actively engaged in team reflexivity during the project. A comprehensive process of scanning existing literature and a scoping survey of key stakeholders providing multiple perspectives informed an extensive program of interviews and consultation. Purposive quota sampling ensured representation of interview participants across key stakeholder domains. It is possible that interview participants may have completed the scoping survey and been influenced by the questions in the survey, however the survey stage closed approximately 2 months prior to the interview stage so it is not anticipated that survey participation would have had an undue influence on interview responses.

Project findings were shared with and informed by a project steering group to confirm transferability across the health, disability, aged care and VET contexts. The inclusion of Victorian participants only may limit the transferability of the findings of this study beyond the Victorian context, however the findings of our study are consistent with the work of others investigating the support workforce in other Australian and international contexts. While all sectors (healthcare, disability, aged care and VET) were represented at each stage of data collection and on the project steering committee, participants in this study were primarily from the healthcare sector. It is possible that more participants from the alternate sectors may have introduced new information, however post-interview debriefing and consultation with the project steering committee indicated that this was unlikely.

## Conclusion

This study sought to explore the factors which may contribute to the promotion of optimal utilisation of AHAs across health, aged care and disability sectors. We identified four categories: system factors, training factors, individual factors and workplace factors. These findings informed a new conceptualisation of AHA career pathway elements that may be important for optimising the utilisation of the AHA workforce across sectors. The findings of this study are based on the views of key stakeholders from Victoria, so further investigation is required to explore the transferability of these findings to national and global contexts, and to further test the applicability of the elements identified in the conceptual model.

## Supplementary Information

**Additional file 1.**

## Data Availability

The raw data from this study is not available as participants did not provide consent for data sharing.

## Abbreviations

AHA: Allied health assistant
AHP: Allied health professional
COREQ: Consolidated Criteria for Reporting of Qualitative Research
VET: Vocational education and training
VAWM: Victorian Assistant Workforce Model
NDIS: National Disability Insurance Scheme
