# Supplementary material for: Exploring utilisation of the allied health assistant workforce in the Victorian health, aged care and disability sectors
Source: BMC Health Serv Res. 2021 Oct 23;21:1144. doi: 10.1186/s12913-021-07171-z (PMC8540135; doi:10.1186/s12913-021-07171-z)
Supplement: Supplementary file 1 — Additional file 1. [file 12913_2021_7171_MOESM1_ESM.pdf]

Additional File 1:

Supplementary Figure 1: Literature scan results

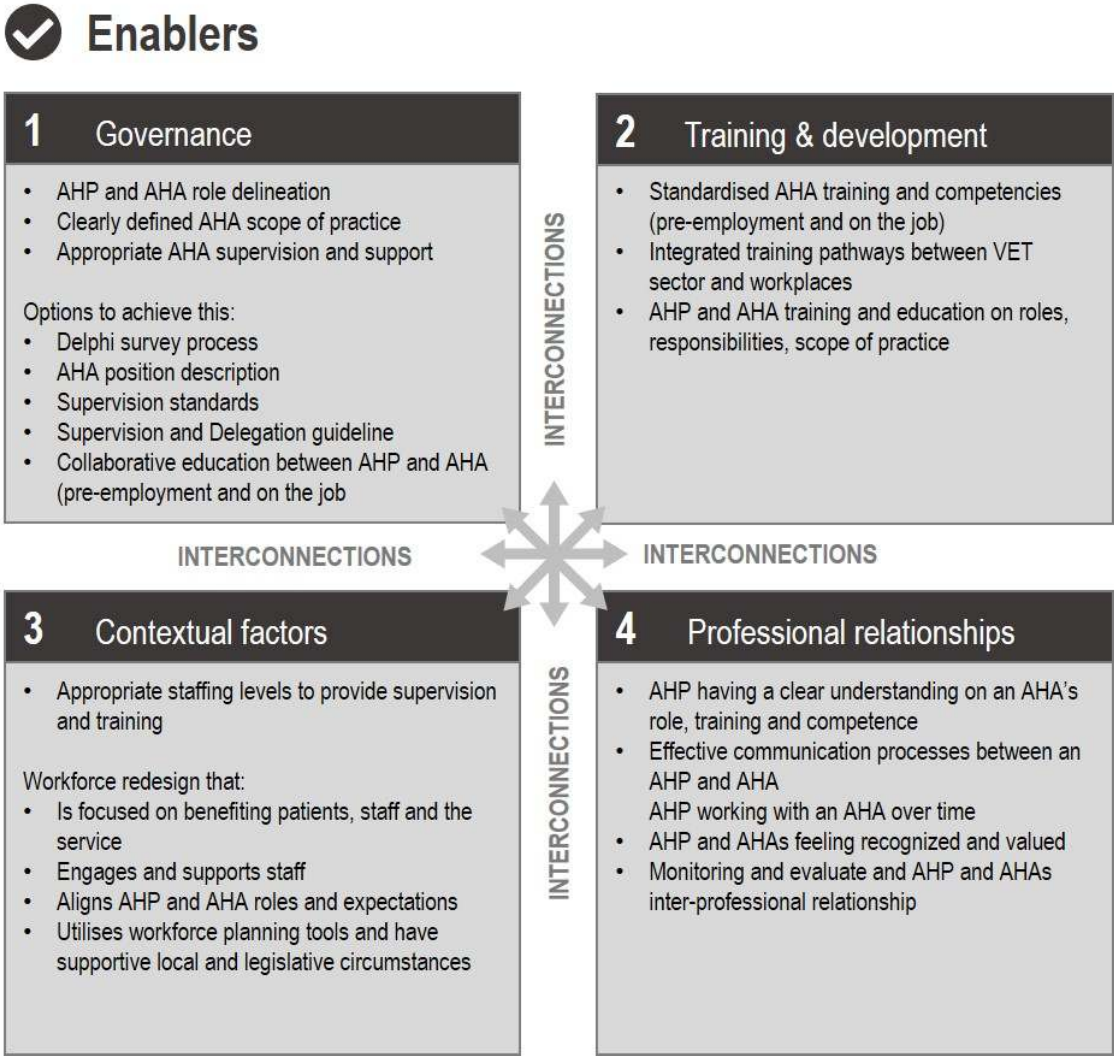

Barriers

1 Governance

- Inconsistencies in an AHA's knowledge and skills
- Government policy documents are not specific enough
- No requirements for a formal qualification/training for an entry level AHA role
- Professional registration and industrial agreements reinforce traditional ways of working

Leads to:

- Inconsistent interpretation of AHA scope of practice
- Limited transferability of role across settings and location
- Blurring of role boundaries between an AHP and an AHA
- AHA role having competing demands between clinical and non clinical tasks
- Lack of recognition and career opportunities for an AHA

2 Training & development

- Lack of evidence to support AHA training and competency requirements
- Inconsistency in AHA training
- Lack of training pathway for AHAs
- AHA pre-employment curriculum has insufficient training in technical skills and across AH discipline, and duration of clinical placement
- AHA on the job training is shaped by the local context and is resource intensive
- AHPs have limited time to supervise and train AHAs
- Time is required for an AHA to reach a proficient skill level
- AHPs undergraduate curriculum does not routinely include supervision and delegation training
- AHPs lacking knowledge in an AHA's role and scope of practice
- AHPs lacking knowledge in supervision and delegation

INTERCONNECTIONS

INTERCONNECTIONS

INTERCONNECTIONS

INTERCONNECTIONS

3 Contextual factors

- Less availability of AHPs to train and supervise AHAs in regional and rural areas
- AHA scope is more restricted in rural and regional areas (role is more generalist and > non-clinical work)
- AHP and AHA staffing levels/shortages
- AHA role shaped by service need thereby contributing to role diversity
- Inadequate workforce redesign processes: insufficient task analysis of an AHP role; insufficient staff engagement; economic driver perceived as the driver for change and poor communication of the AHA role and vision

4 Professional relationships

- AHP with no previous experience working with an AHA
- AHP uncertainty of an AHA's role, training and skills
- AHP perceived role threat (devalued)
- AHP perceived decreased job satisfaction
- AHP perceives job insecurity (role substitution) leading to AHP reluctance to delegate to an AHA

Supplementary Table 1a: Demographics of survey respondents by Work Location and Work Role

		Metropolitan				Rural or Remote			
		AHAs		AHPs and AHA Leaders		AHAs		AHPs and AHA Leaders	
		Count	N %	Count	N %	Count	N %	Count	N %
Respondent age category	18-24	15	65.2%	8	34.8%	7	70.0%	3	30.0%
	25-34	52	28.1%	133	71.9%	16	29.1%	39	70.9%
	35-44	53	35.1%	97	64.2%	13	26.0%	35	70.0%
	45-54	43	38.7%	66	59.5%	23	53.5%	19	44.2%
	55-64	33	51.6%	30	46.9%	23	69.7%	9	27.3%
	65+	3	75.0%	1	25.0%	0	0.0%	1	100.0%
	Totals	*199*	*37.1%*	*335*	*62.2%*	*82*	*42.5%*	*106*	*55.4%*
Respondent gender	Female	167	35.5%	299	63.6%	73	43.2%	92	54.4%
	Male	30	50.0%	30	50.0%	7	33.3%	14	66.7%
	Non-binary or not specified	1	50.0%	1	50.0%	0	0.0%	1	100.0%
	Totals	*198*	*37.1%*	*330*	*62.2%*	*80*	*42.5%*	*107*	*55.4%*
Respondent Residency status	Australian Citizen	132	35.3%	238	63.6%	53	44.5%	64	53.8%
	Permanent Resident	64	40.0%	96	60.0%	29	39.7%	42	57.5%
	Temporary Visa Holder	3	100.0%	0	0.0%	0	0.0%	0	0.0%
	Other	1	100.0%	0	0.0%	0	0.0%	0	0.0%
	Totals	*200*	*37.1%*	*334*	*62.2%*	*82*	*42.5%*	*106*	*55.4%*
Highest Education Level	High School Certificate	5	100.0%	0	0.0%	0	0.0%	0	0.0%
	Certificate III	7	100.0%	0	0.0%	4	100.0%	0	0.0%
	Certificate IV	75	98.7%	1	1.3%	36	97.3%	0	0.0%
	Associate Diploma	5	100.0%	0	0.0%	1	100.0%	0	0.0%
	Division 2 Nurse	21	84.0%	4	16.0%	15	83.3%	1	5.6%
	Division 2 Nurse	0	0.0%	0	0.0%	7	100.0%	0	0.0%
	Division 1 Nurse	2	100.0%	0	0.0%	3	100.0%	0	0.0%
	Bachelor Degree	56	29.5%	133	70.0%	11	14.9%	63	85.1%
	Post Graduate Certificate	5	13.5%	32	86.5%	0	0.0%	5	100.0%
	Post Graduate Diploma	10	30.3%	22	66.7%	4	36.4%	7	63.6%
	Masters Degree	12	8.5%	127	90.1%	1	3.2%	30	96.8%
	PhD	0	0.0%	16	100.0%	0	0.0%	1	100.0%
	Totals	*198*	*37.1%*	*335*	*62.2%*	*82*	*42.5%*	*107*	*55.4%*
Years of experience	Less than 1 year	20	83.3%	4	16.7%	6	50.0%	6	50.0%
	1-5 years	72	52.6%	65	47.4%	35	56.5%	24	38.7%
	6-10 years	53	41.4%	75	58.6%	14	50.0%	14	50.0%
	Greater than 10 years	55	22.2%	191	77.0%	27	30.0%	62	68.9%
	Totals	*200*	*37.1%*	*335*	*62.2%*	*82*	*42.5%*	*106*	*55.4%*
Number of AH roles	1	65	54.6%	54	45.4%	32	60.4%	20	37.7%
	2	51	55.4%	41	44.6%	19	45.2%	21	50.0%
	3	37	43.5%	48	56.5%	13	43.3%	17	56.7%
	4	12	20.3%	47	79.7%	5	27.8%	13	72.2%
	5	9	17.3%	43	82.7%	5	31.3%	11	68.8%

	6	5	15.2%	28	84.8%	2	50.0%	2	50.0%
	7	2	20.0%	8	80.0%	1	14.3%	5	71.4%
	8	0	0.0%	13	100.0%	0	0.0%	1	100.0%
	9	0	0.0%	5	100.0%	1	100.0%	0	0.0%
	10 or more	12	23.1%	39	75.0%	2	11.8%	15	88.2%
	Totals	*193*	*37.1%*	*326*	*62.2%*	*80*	*42.5%*	*105*	*55.4%*
Years at current organisation	Less than 1 year	27	47.4%	30	52.6%	9	40.9%	13	59.1%
	1-5 years	93	41.2%	133	58.8%	30	36.1%	50	60.2%
	6-10 years	47	42.3%	64	57.7%	17	51.5%	16	48.5%
	Greater than 10 years	32	22.5%	108	76.1%	26	48.1%	27	50.0%
	Totals	*199*	*37.1%*	*335*	*62.2%*	*82*	*42.5%*	*106*	*55.4%*
Intention to stay	Less than 1 year	9	75.0%	3	25.0%	6	66.7%	3	33.3%
	1-3 years	66	63.5%	38	36.5%	16	50.0%	15	46.9%
	4-5 years	47	49.0%	49	51.0%	18	51.4%	17	48.6%
	6-10 years	26	29.9%	59	67.8%	23	52.3%	20	45.5%
	Greater than 10 years	50	21.4%	184	78.6%	18	25.4%	51	71.8%
	Totals	*198*	*37.1%*	*333*	*62.2%*	*81*	*42.5%*	*106*	*55.4%*

Supplementary Table 1b: Work setting and Disciplines worked with by Work Role and Work location

	Metropolitan		Rural or Remote	
	AHAs	AHPs and AHA Leaders	AHAs	AHPs and AHA Leaders
	Count	Count	Count	Count
**Work setting***				
Subacute inpatients	75	99	27	38
Acute inpatients	60	133	32	51
Community Health	38	63	39	43
Hydrotherapy	24	16	21	16
Health independence program services e.g. postacute care (PAC), subacute ambulatory care services (SACS), Complex Care	22	38	17	22
Private Hospital Inpatients	17	5	3	1
Aged Care Residential	16	39	27	30
Children's' Allied Health Service	10	15	14	9
Private Hospital Outpatients	10	7	2	2
Not-for-profit sector	9	19	3	9
NDIS provider community	7	33	17	27
Disability sector	7	15	8	10
Acute outpatients	7	44	15	19
Home and Community Care for Younger People	5	12	7	14
Allied Health Private Practice - NDIS	4	18	4	9
Complex Care	4	10	4	6
NDIS provider residential	2	8	2	1
Primary Care Services	2	9	11	14
Allied Health Private Practice - Aged Care	1	4	0	5
Aged Care Assessment Service	1	2	1	0
School	0	0	0	5
Community palliative care	0	0	1	5
Lymphoedema	0	3	8	4
Subacute outpatients	0	0	0	0
NDIS Positive Behaviour Support Provider	0	2	1	1
**Disciplines worked with***				
Art Therapy	3	15	1	1
Audiology	2	16	2	8
Behaviour Support Practitioner	3	7	1	4
Dietetics/Nutrition	51	139	33	54
Exercise Physiology	40	55	32	33
Music Therapy	3	33	0	1
Neuropsychology	7	81	3	11
Occupational Therapy	114	190	67	72
Orthotics	4	52	2	16
Pastoral Care (incl. chaplaincy)	2	41	0	6
Physiotherapy	154	226	67	74
Play Therapy	2	10	0	2
Podiatry	24	74	21	33
Psychology	13	98	3	22
Social Work	35	138	20	51
Speech Therapy	62	156	45	65
Other (please specify)	0	0	0	0

Supplementary Table 2*: Independent t-test comparing AHA and AHP & Allied health leaders' nomination of AHA Core skills*

Question text: What are the core skills and attributes required of an AHA to be successful in your workplace?

Response format: selected(1)/not selected(0)

	AHAs (n=284)		AHPs & AHA Leaders (n=443)		Total (n=727)				
**Item wording**	**Mean**	**SD**	**Mean**	**SD**	**Mean**	**SD**	**t**	**df**	**p value**
Ability to make sound judgements	0.80	0.40	0.77	0.42	0.78	0.42	1.2	630.5	0.227
Interest in the job	0.86	0.35	0.85	0.36	0.85	0.36	0.5	725.0	0.640
Communication skills	0.99	0.12	0.96	0.19	0.97	0.16	1.9	724.9	0.052
Confidence	0.77	0.42	0.58	0.49	0.65	0.48	5.4	667.2	**<0.001**
Need to be able to able to assert their own role boundaries/competence/confidence	0.79	0.41	0.64	0.48	0.70	0.46	4.2	666.8	**<0.001**
Drive	0.53	0.50	0.36	0.48	0.42	0.49	4.6	584.9	**<0.001**
Have developed the role themselves	0.25	0.43	0.06	0.24	0.13	0.34	6.6	399.7	**<0.001**
Assertiveness	0.46	0.50	0.24	0.43	0.33	0.47	6.3	535.1	**<0.001**
Initiative	0.83	0.37	0.79	0.41	0.80	0.40	1.2	645.6	0.097
Ability to 'think outside the box'	0.74	0.44	0.46	0.45	0.57	0.50	8.1	658.2	**<0.001**
Need for self-direction	0.60	0.49	0.44	0.50	0.50	0.50	4.2	608.9	**<0.001**
Trustworthy (more than just a police check)	0.76	0.43	0.71	0.46	0.73	0.45	1.6	629.1	0.132
Ability to think/reflect on role	0.73	0.44	0.74	0.44	0.74	0.44	-0.4	725.0	0.708
Type of people who will continually improve (e.g. undertake training)	0.68	0.47	0.69	0.46	0.69	0.46	-0.3	725.0	0.779
Experience	0.59	0.49	0.30	0.46	0.41	0.49	8.1	571.3	**<0.001**
Training to underpin competence, formal qualifications	0.62	0.49	0.54	0.50	0.57	0.50	2.1	615.1	0.034
Willing to accept responsibility	0.81	0.39	0.70	0.46	0.74	0.44	3.6	668.9	**<0.001**
Willing to learn	0.89	0.31	0.91	0.28	0.91	0.29	-0.8	725.0	0.430
Clinical competence	0.82	0.38	0.68	0.47	0.74	0.44	4.4	682.7	**<0.001**

Bonferroni correction applied 0.05/19 =0.0026, level of significance set at p<0.002

Supplementary Table 3: Independent t-test comparing AHA and AHP & Allied health leaders' perspectives related to current job role

Question wording: The following statements relate to your current role and workplace. Please mark the following statements according to your level of agreement with each

Response options: Strongly disagree(1), Disagree(2), Neither Agree or Disagree(3), Agree(4), Strongly Agree(5), Not applicable (not scored)

	AHAs				AHPs and AHA Leaders				Total						
**Item wording**	**N**	**Median**	**Mean**	**SD**	**N**	**Median**	**Mean**	**SD**	**N**	**Median**	**Mean**	**SD**	**t**	**df**	**p value**
**Individual items**															
I consider my job very satisfying	282	4	4.29	0.82	435	4	4.42	0.66	717	4	4.37	0.73	-2.2	508.3	0.026
I am satisfied with the initial training and upskilling offered	282	4	3.74	1.03	431	4	4.02	0.82	713	4	3.91	0.92	-3.9	504.7	**<0.001**
I have adequate ongoing training and development to meet my needs	281	4	3.54	1.09	435	4	4.04	0.85	716	4	3.84	0.98	-6.5	492.1	**<0.001**
I am satisfied that my job role has sufficient career development opportunities	282	3	2.82	1.26	434	4	3.75	1.08	716	4	3.38	1.24	-10.1	536.1	**<0.001**
I find my daily work stimulating	283	4	3.96	0.86	432	4	4.26	0.68	715	4	4.14	0.77	-5.2	713	**<0.001**
I have opportunities to lead quality initiatives	279	4	3.41	1.05	429	4	4.10	0.92	708	4	3.83	1.03	-9.0	539	**<0.001**
I have opportunities to lead research initiatives	274	3	2.73	1.05	424	4	3.44	1.12	698	3	3.16	1.14	-8.4	696	**<0.001**
I have opportunities to be involved with quality initiatives	275	4	3.49	1.07	428	4	4.15	0.87	703	4	3.89	1.01	-8.6	502	**<0.001**
I have opportunities to be involved with research initiatives	274	3	3.00	1.12	430	4	3.54	1.11	704	4	3.33	1.15	-6.4	702	**<0.001**
I have opportunities to supervise students	273	4	3.70	1.18	409	4	4.17	1.04	682	4	3.98	1.12	-5.3	532.5	**<0.001**

Bonferroni correction applied 0.05/10 =0.005, level of significance set at p<0.005

	AHAs				AHPs and AHA Leaders				Total						
**Item wording**	**N**	**Median**	**Mean**	**SD**	**N**	**Median**	**Mean**	**SD**	**N**	**Median**	**Mean**	**SD**	**t**	**df**	**p value**
**Workplace items**															
AHA scope of practice is well defined in my workplace	282	4	3.72	1.09	417	4	3.69	1.02	699	4	3.70	1.05	0.4	697	0.645
All AHAs are optimally utilised in my workplace	279	4	3.38	1.25	417	3	3.10	1.10	696	3	3.21	1.17	3.0	543	**0.002**
AHA clinical supervision (formal) needs are adequately met in my workplace	280	4	3.69	1.18	395	4	3.42	1.00	675	4	3.53	1.09	3.0	539	**0.002**
AHA clinical supervision (informal) needs are adequately met in my workplace	280	4	3.86	1.05	402	4	3.66	0.93	682	4	3.74	0.99	2.6	680	**0.009**
AHA training is guided by a structured framework in my work place	281	3	3.31	1.18	403	3	3.30	1.04	684	3	3.30	1.10	0.1	555	0.893

Bonferroni correction applied 0.05/5 =0.01, level of significance set at p<0.01

Supplementary Table 4: Participant profile of interview participants

Category	Number of groups	Total participants
**Focus group**		
AHA (metro)	3	26
AHA (regional)	2	15
AHPs & Leaders (metro)	3	19
AHPs & Leaders (regional)	2	13
AHA students	1	5
VET sector educators & managers	1	12
Consumers	1	4
**Individual**		
Professional association representatives	n/a	19
Health sector representatives	n/a	3
Disability sector representatives	n/a	2
Consumer	n/a	1

Supplementary Table 5. Analytic process for "Individual" category

Illustrative data	Sub themes with	Main Theme	Category
"I've seen ones [AHAs] who came in who have no idea what Allied Health really was when they start because they come from other roles. So, for example, we currently have an AHA now who's never worked in health before. She was an admin person in the council. So we've really had to put in a lot of effort to give her the training to be able to do things with the client in terms of goal setting and all that sort of thing as well and how equipment worked and how OTs even come to our conclusions or recommendations, so starting from scratch that way.<br>But then again, we also have AHAs who are studying physio, for example, and working part-time as an AHA. So, they're all over it all. So, it's a real range that we're seeing." ***[AHA, Focus Group, regional]***	AHA or student's life experience<br>AHA or student's personal attributes	AHA personal attributes prior to entering workforce	Individual
"I think our AHA's have got quite a good grounding and they're very much aware of their limitations and got a very good grip on what's appropriate and what's not and when to feedback and when not to" ***[AHP, Focus Group, metro]***<br>"…we have some very experienced AHAs at our workplace and we recognise their scope of practice based on their skills. So, they might be doing things I guess at a higher level than what we would expect someone coming in to the profession. Say if someone's worked in an area for five years or more, naturally they're more independent in their role and they're doing things more independently than what we'd expect of someone new." ***[AHP, Focus Group, metro]***<br>"It's an area that we can definitely be used. I think people just need to have a bit more open mind and not to be worried that we're going to take their job. We're just here to help within the workforce and develop our skills because sometimes, some of the feedback from some therapists, they are afraid that we're progressing and may take their job and that's not the case. All of us love being an allied health assistant, that's why we're within this industry for a long time. We just want to grow and develop as they do." ***[AHA, Focus Group, metro]***	AHA ability to learn new skills<br>AHA ability to manage multiple competing demands<br>AHA ability to recognise & feedback client issues<br>AHA attitude to learning new skills<br>AHA confidence in AHP influenced by AHP grade & experience<br>AHA knowledge & self-advocacy for SoP<br>AHA networking<br>AHA role to initiate and drive CPD<br>AHA skills fit for purpose<br>AHA value of an ongoing learning culture for an AHA role	AHA personal attributes in the workplace	
